# Network segregation in aging females and evaluation of the impact of sex steroid hormones

**DOI:** 10.3389/fnhum.2023.1059091

**Published:** 2023-02-01

**Authors:** Tracey H. Hicks, Thamires N. C. Magalhães, Hannah K. Ballard, T. Bryan Jackson, Sydney J. Cox, Jessica A. Bernard

**Affiliations:** ^1^Department of Psychological and Brain Sciences, Texas A&M University, College Station, TX, United States; ^2^Texas A&M Institute for Neuroscience, Texas A&M University, College Station, TX, United States

**Keywords:** functional connectivity, aging, steroid hormones, sex differences, network segregation

## Abstract

Males and females show differential patterns in connectivity in resting-state networks (RSNs) during normal aging, from early adulthood to late middle age. Age-related differences in network integration (effectiveness of specialized communication at the global network level) and segregation (functional specialization at the local level of specific brain regions) may also differ by sex. These differences may be due at least in part to endogenous hormonal fluctuation, such as that which occurs in females during midlife with the transition to menopause when levels of estrogens and progesterone drop markedly. A limited number of studies that have investigated sex differences in the action of steroid hormones in brain networks. Here we investigated how sex steroid hormones relate to age-network relationships in both males and females, with a focus on network segregation. Females displayed a significant quadratic relationship between age and network segregation for the cerebellar-basal ganglia and salience networks. In both cases, segregation was still increasing through adulthood, highest in midlife, and with a downturn thereafter. However, there were no significant relationships between sex steroid hormone levels and network segregation levels in females, and they did not exhibit significant associations between progesterone or 17β-estradiol and network segregation. Patterns of connectivity between the cerebellum and basal ganglia have been associated with cognitive performance and self-reported balance confidence in older adults. Together, these findings suggest that network segregation patterns with age in females vary by network, and that sex steroid hormones are not associated with this measure of connectivity in this cross-sectional analysis. Though this is a null effect, it remains critical for understanding the extent to which hormones relate to brain network architecture.

## 1. Introduction

Advanced age is associated with an overall decrease in the effectiveness of specialized communication at the global network level (i.e., integration) and loss of functional specialization at the local level of specific brain regions (i.e., segregation). That is, neuronal networks become less distinct with advanced age (Foo et al., 2021).

Age-related differences in the reorganization of functional connectivity and cognitive abilities may also differ by sex. In adulthood, sex differences in brain structure and function can be observed (Cosgrove et al., 2007) and, similarly, sex differences in resting state networks (RSNs) have been reported. For example, studies of age differences have observed that males show increasing connectivity between-networks when compared to females (Allen et al., 2011; Filippi et al., 2013; Goldstone et al., 2016). Males also exhibited more marked changes in default mode network (DMN) connectivity, especially in the posterior cingulate cortex (PCC), but showed smaller differences (and possibly increases) in connectivity to the lateral prefrontal regions of the fronto-parietal network (FPN) relative to females (Allen et al., 2011; Filippi et al., 2013). Females on the other hand showed smaller differences in DMN connectivity but showed greater decreases in FPN connectivity when compared to males.

Sex differences in network architecture have also been reported (Zhang et al., 2016). Zhang et al. (2016) observed that female functional networks have significantly more connected nodes than males suggest an increase in network homogeneity in female brains. They also observed that the cerebellar nodes have a higher clustering coefficient and local efficiency for females (Zhang et al., 2016). This adds evidence to their findings that the clustering coefficient and local efficiency in males are higher, while female connections are diffuse across lobes and the network is less modular. These results jointly support the notion that networks in female brains, compared to those in males, are more spatially distributed but with lower correlation strengths (Scheinost et al., 2015; Zhang et al., 2016). Overall, males and females showed differential patterns in connectivity in RSNs during normal aging, and from early adulthood to late middle age (Allen et al., 2011; Filippi et al., 2013; Scheinost et al., 2015). However, these differences vary between studies with respect to regions and networks that are impacted.

Just as there are mixed results regarding sex differences with age in brain networks, there are also disagreements in the literature regarding differences in resting-state functional connectivity in the context of hormonal differences between biological sexes. To this point, there have been a limited number of studies that have investigated sex steroid hormones on brain networks. Further, there is currently disagreement among the rapidly expanding number of studies on the possible neuroprotective effects of sex hormones on cognitive and brain function more generally (Moffat, 2005; Sundström Poromaa and Gingnell, 2014; Toffoletto et al., 2014). Given that RSNs appear to be differentially impacted in males in females in later life, further exploration of the impact of sex steroid hormone levels on network architecture across adulthood stands to improve our understanding of underlying factors contributing to these differences. Specifically, it may be that sex hormone levels are related to the integration and segregation of neuronal networks (Peper et al., 2011).

In the brain, hormone receptors can be found across several regions. Post-mortem studies have found estrogen receptors in the hippocampus, claustrum, cerebral cortex, amygdala, hypothalamus, subthalamic nucleus, and thalamus (Osterlund et al., 2000; Weiser et al., 2008; Syan et al., 2017). As for progesterone, a post-mortem study reported high concentrations of its receptors in the amygdala, hypothalamus, and cerebellum (Bixo et al., 1997; Syan et al., 2017). Testosterone exerts an early organizational effect on the development of the hypothalamus (Jacobson et al., 1981), the cerebral cortex (Diamond, 1991), and the hippocampus (Roof and Havens, 1992) in addition to other brain structures. With respect to testosterone, after conversion to estradiol, it can also interact with estrogen receptors (Moffat, 2005). The relationship between these two hormones (testosterone and estrogen) have been shown to affect vascular health directly (Aggarwal et al., 2018; Raparelli et al., 2022). The ratio of testosterone to estradiol is important for vascular function suggesting that these hormones may not act independently (van Koeverden et al., 2019; Raparelli et al., 2022). Therefore, it is also important to investigate the interactions that may occur between sex hormones and whether together they may be involved related to aging processes, as well as differences in neuronal networks (Syan et al., 2017). Testosterone, progesterone, and estrogens are present in both males and females, but their levels and production vary, mainly with respect to sex and age, though fluctuations in estrogens and progesterone occur across the female menstrual cycle as well (Sundström Poromaa and Gingnell, 2014).

The influence of sex hormones on functional networks is vital to our understanding of brain function and organization during periods of endogenous hormonal fluctuation, such as that which occurs in females during midlife with the transition to menopause when levels of estrogens and progesterone drop markedly (Toffoletto et al., 2014; Foo et al., 2021). Our study here aims to investigate how sex steroid hormones relate to age-network relationships in both males and females, with a focus on network segregation. As such, we predicted that there would be associations between network segregation and hormone levels, as well as interactions between hormones that may be affecting or enhancing the segregation of RSNs.

## 2. Materials and methods

### 2.1. Study sample

One hundred and fifty-seven participants (total *n* = 157) were enrolled as part of a larger study on aging. All participants underwent a battery of cognitive and motor tasks and during this assessment, the participants provided saliva samples for hormone quantification (for details about collection see Section “2.2 Hormone quantification”). After the behavioral visit the participants returned for a magnetic resonance imaging (MRI) session approximately 2 weeks later. However, due to unexpected delays related to the COVID-19 pandemic, the time between the two sessions (39.0 days ± 21.4 days) varied between participants. For our analyses here, we focused only on the hormone and brain imaging data.

**FIGURE 1:**
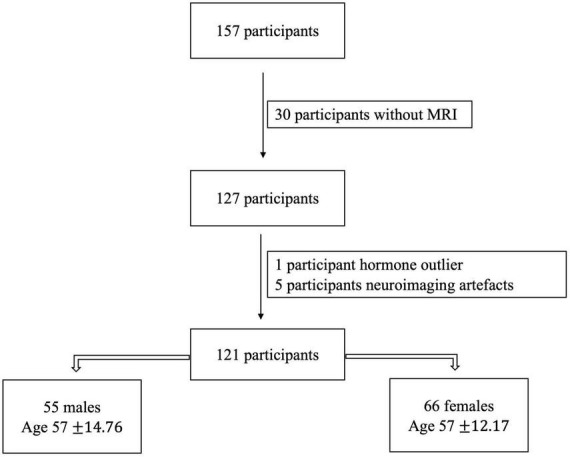
The initially collected number of participants and after following the exclusion criteria, the final number of our sample.

Exclusion criteria were history of neurological disease, stroke, or formal diagnosis of psychiatric illness (e.g., depression or anxiety), contraindications for the brain imaging environment, and use of hormone therapy (HTh) or hormonal contraceptives [intrauterine device (IUD), possible use of continuous birth control (oral), and history of hysterectomy]. These latter exclusions were made to evaluate impacts of normative endocrine aging on healthy adult females. For our analyses here we focused only on those with available neuroimaging data and hormonal assays. Thus, our final sample included 121 participants [55 males (age 57 ± 14.76) and 66 females (age 57 ± 12.17)]. A flowchart showing the exclusions and determination of the final sample for analysis is presented below (Figure 1).

All study procedures were approved by the Institutional Review Board at Texas A&M University, and written informed consent was obtained from each participant prior to initiating any data collection.

### 2.2. Hormone quantification

For hormonal analyses, we followed the methodology used in recent work from our research group (Ballard et al., 2022a). For the sake of clarity and replicability, the methods reporting here matches what was reported in our recent work (Ballard et al., 2022a). Before collecting the saliva sample, participants were asked to refrain from consuming alcohol 24 h prior and eating or drinking 3 h prior to their first study session to avoid exogenous influences on hormone levels. Participants were also screened for oral disease or injury, use of substances such as nicotine or caffeine, and prescription medications that may impact the saliva pH and compromise samples. Participants were asked to rinse their mouth with water 10 min prior to providing a saliva sample to clear out any residue.

Samples were then collected in pre-labeled cryovials provided by Salimetrics^1^ using the passive drool technique. For our study, participants were asked to supply 1 mL of saliva, after which samples were immediately stored in a −80° Celsius bio-freezer for stabilization. Assays were completed by Salimetrics to quantify 17β-estradiol, progesterone, and testosterone levels for each participant. The amount of saliva collected was sufficient to detect 17β-estradiol at a high sensitivity threshold of 0.1 pg/mL (Salimetrics, 2022), along with 5.0 pg/mL and 1.0 pg/mL thresholds for progesterone and testosterone, respectively.

The protocol used by Salimetrics includes two repetitions of each assay; thus, the values used in our analyses represent an average of both repetitions. A few samples were insufficient in quantity and were unable to be properly assayed (*n* = 3; 2 progesterone, 1 testosterone). The intra-assay coefficient of variability for our hormone samples was 0.15 for 17β-estradiol, 0.11 for progesterone, and 0.07 for testosterone. This non-invasive method is adequate for precisely measuring reproductive hormones. Salivary measurements are strongly correlated with blood-derived measurements to index sex hormone levels (17β-estradiol: *r* = 0.80; progesterone: *r* = 0.80; testosterone: *r* = 0.96).^2^

### 2.3. Imaging acquisition

Participants underwent structural and resting-state MRI using a Siemens Magnetom Verio 3.0 Tesla scanner and a 32-channel head coil. For structural MRI, we collected a high-resolution T1-weighted 3D magnetization prepared rapid gradient multi-echo (MPRAGE) scan [repetition time (TR) = 2,400 ms; acquisition time = 7 min; voxel size = 0.8 mm^3^] and a high-resolution T2-weighted scan [TR = 3,200 ms; acquisition time = 5.5 min; voxel size = 0.8 mm^3^], each with a multiband acceleration factor of two. For resting-state imaging, we administered four blood-oxygen level dependent (BOLD) functional connectivity (fcMRI) scans with the following parameters: multiband factor of eight, 488 volumes, TR of 720 ms, and 2.5 mm^3^ voxels. Each fcMRI scan was 6 min in length for a total of 24 min of resting-state imaging, and scans were acquired with alternating phase encoding directions (i.e., two anterior to posterior scans and two posteriors to anterior scans). During the fcMRI scans, participants were asked to lie still with their eyes open while fixating on a central cross. In total, the acquisition of images takes about 45 min, including a 1.5-min localizer.

Scanning protocols were adapted from the multiband sequences developed by the Human Connectome Project (HCP) (Harms et al., 2018) and the Center for Magnetic Resonance Research at the University of Minnesota to facilitate future data sharing and reproducibility.

#### 2.3.1. Imaging processing

##### 2.3.1.1. Pre-processing

Images were converted from DICOM to NIFTI and organized into a Brain Imaging Data Structure (BIDS, version 1.6.0) format *via* the latest docker container version of bidskit (version 2021.6.14).^3^ Using the split tool distributed with the FMRIB Software Library (FSL) package (Jenkinson et al., 2012), a single volume was extracted from two oppositely encoded BOLD images to estimate B_0_ field maps. Next, fMRIPrep (version 20.2.3)^4^ was used to preprocess anatomical and functional images. The fMRIPrep preprocessing pipeline includes basic steps such as co-registration, normalization, unwarping, noise component extraction, segmentation, and skull-stripping.

While basic pre-processing was performed in the fMRI preparation, we also completed remaining steps in the Conn toolbox, version 21a (Whitfield-Gabrieli and Nieto-Castanon, 2012). We used the default preprocessing pipeline, which consists of realignment and unwarping with motion correction, centering to (0, 0, 0) coordinates, slice-timing correction, outlier detection using a 95th percentile threshold and the Artifact Rejection Toolbox (ART), segmentation of gray matter, white matter, and cerebrospinal fluid, normalization to Montreal Neurological Institute (MNI) space, and spatial smoothing with a 5 mm full width at half-maximum (FWHM) Gaussian kernel. A band-pass filter of 0.008–0.099 Hz was applied to denoise data. The threshold for global-signal *z*-values was set at three, while the motion correction threshold was set at 0.5 mm. After being de-spiked during denoising to adhere to the global mean, 6-axis motion data and frame-wise outliers were included as first-level covariates.

##### 2.3.1.2. Region of interest selection

In our study, we used the same Region of Interest (ROIs) selection used by Ballard et al. (2022c), in work previously completed by our group. The MNI coordinates for each cortical node retrieved from Cassady et al. (2019) [originally derived from Power et al. (2011)] were selected by our group. Further, we included 20 subcortical nodes of the [extracted from Hausman et al. (2020)] for the cerebellar-basal ganglia network (Diedrichsen, 2006; Di Martino et al., 2008; Diedrichsen et al., 2009). Cerebellar seeds were determined *via* the SUIT atlas (Power et al., 2011; Cassady et al., 2019). Our final set of ROIs contained 234 nodes across 11 networks (10 cortical, 1 subcortical, see Table 1 for a list of included networks). MNI coordinates for each node were translated to voxel coordinates, which were subsequently used to create spherical seeds with 3.5 mm diameters in FSL (Jenkinson et al., 2012). These seeds were then treated as ROIs.

**TABLE 1:** Network abbreviation key.

Abbreviation	Network
Au	Auditory
CBBG	Cerebellar-basal ganglia
COTC	Cingulo-opercular task control
DA	Dorsal attention
DM	Default mode
FPTC	Fronto-parietal task control
Sa	Salience
SSH	Sensory somatomotor hand
SSM	Sensory somatomotor mouth
Vi	Visual
VA	Ventral attention

First-level ROI-to-ROI relationships were evaluated with a bivariate correlation approach; those correlations are needed to calculate the variables within and between the network for each subject which are then used in the network segregation equation. Correlation values were transformed into *z*-values *via* Fisher’s r-to-z conversion (Zar Jerrold, 1996). Corrections for multiple comparisons were applied during statistical analyses.

##### 2.3.1.3. Network segregation equation

For the analysis of network segregation, we again followed our previous work reported by Ballard et al. (2022a,c) and based off analyses initially conducted by Chan et al. (2014). Network segregation values were determined using Equation 1 below. In the formula, ${\underline{z}}_{w}$ corresponds to the mean correlation between ROIs within an individual network, and ${\underline{z}}_{b}$ represents the mean correlation between ROIs of an individual network and all remaining ROIs of other networks. Group-level analyses were performed with a voxel threshold of *p* < 0.001 and cluster threshold, FDR-corrected, of *p* < 0.05.

Equation 1. Network segregation values were determined using this formula.


$$N\,e\,t\,w\,o\,r\,k\,s\,e\,g\,r\,e\,g\,a\,t\,i\,o\,n=\frac{{\underline{z}}_{w}-{\underline{z}}_{b}}{{\underline{z}}_{w}}$$

### 2.4. Statistical analysis

We first sought to ascertain sex differences in hormone levels within our sample. Analyses of variance (ANOVAs) were conducted to determine sex differences in hormone levels (i.e., estradiol, progesterone, and testosterone separately). ANOVAs were completed using the ANOVA function from the default “stats” package in R (v4.0.5, R Core Team, 2021) which determined beta coefficients, degrees of freedom, *F*-values, and *p*-values; the sjstats and pwr packages were used to compute η^2^ and partial η^2^ values (v0.18.1, (Lüdecke, 2022)).

As our primary area of interest lies within the impact of fluctuating hormones on female brain network segregation, our main analyses investigated females only. However, exploratory analyses evaluated males and all participants combined which are included in the supplement.

To explore the unique associations between hormone levels (i.e., estradiol, progesterone, and testosterone separately) and each network of interest in females, linear regressions were performed in which hormone levels served as the predictor and network segregation as the outcome. These linear regressions were also conducted in exploratory analyses with males and all participants collapsing the two sexes (Supplementary Tables 1–6). The lm function from the default “stats” package in R (v4.0.5, R Core Team, 2021) determined beta coefficients, degrees of freedom, *F*-values, *p*-values, R^2^, and adjusted R^2^. False discovery rate (FDR) correction was applied to account for multiple comparisons (i.e., number of networks examined) using the FSA package in R exclusively on results with a *p*-value ≤ 0.05 (FSA v0.9.3, Ogle et al., 2022). Linear regressions with hormone level interactions (i.e., estradiol*progesterone, estradiol*testosterone, and progesterone*testosterone) as the predictor variables explored the relationship between combined hormone levels and network segregation in females. Exploratory analyses investigated the same hormone level interactions in males and all participants together (Supplementary Tables 12, 13). FDR correction was applied as previously described (FSA v0.9.3, Bernard et al., 2015). Our cross-sectional hormone level data is also visualized *via* locally weighted scatterplot smoothing (Supplementary Figure 1) to provide comparison to normal distributions of sex hormone levels by age.

Associations between network segregation and age were evaluated *via* linear regression with age as the predictor and network connectivity as the outcome in females; males and all participants together were run as exploratory analyses (Supplementary Tables 7, 8). We conducted similar regressions with quadratic age [Age + I (Age^2^)] as the predictor to investigate whether network segregation demonstrated better fit with a quadratic function rather than linear across middle-to advanced-age adults, given prior work suggesting non-linear relationships between brain system segregation (Chan et al., 2014) as well as brain volume (Bernard et al., 2015) and age. Quadratic regressions for males and all participants were run as exploratory analyses (Supplementary Tables 9, 10). Linear and quadratic regressions were performed using the lm function from the default “stats” package in R (v4.0.5, R Core Team, 2021) which determined beta coefficients, degrees of freedom, F-values, *p*-values, R^2^, and adjusted R^2^. FDR correction was applied as previously described to account for multiple comparisons (i.e., number of networks examined) (FSA v0.9.3, Chan et al., 2014). Akaike’s An Information Criterion (AIC) (Sakamoto et al., 1986) compared fit between linear and quadratic models with a requirement that model value must differ by 10 to be considered a superior fit (Burnham and Anderson, 2004; Bohon and Welch, 2021). AIC was calculated by the default “stats” package in R (v4.0.5, R Core Team, 2021).

**FIGURE 2:**
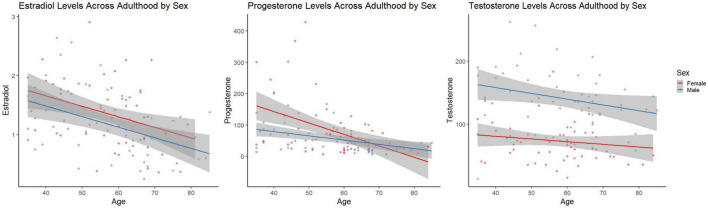
Linear scatter plots demonstrate estradiol levels in females and males across adulthood. The gray superimposed on each colored line depicts the 95% confidence interval in each sex. Estradiol levels **(left)** were not significantly different by sex when accounting for age (*p* = 0.071). Progesterone levels **(middle)** were not significantly different by sex when accounting for age (*p* = 0.073). Testosterone levels **(right)** were significantly different by sex when accounting for age (*p* < 0.001).

**FIGURE 3:**
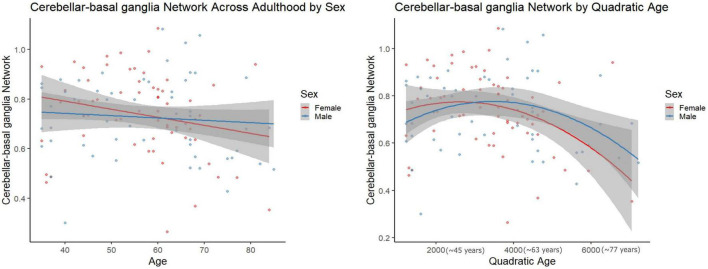
Scatter plots demonstrate the linear **(left)** and quadratic **(right)** relationship between Cerebellar-basal ganglia network connectivity and age. Parentheses next to quadratic values **(right)** indicate the approximate square root of the quadratic value for interpretative purposes. The gray superimposed on each colored line depicts the 95% confidence interval in each sex.

**FIGURE 4:**
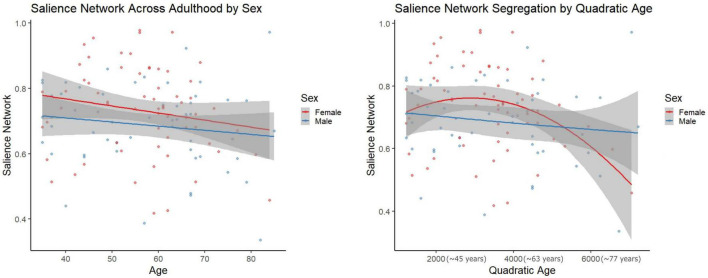
Scatter plots demonstrate the linear **(left)** and quadratic **(right)** relationship between salience network connectivity and age. Parentheses next to quadratic values **(right)** indicate the approximate square root of the quadratic value for interpretative purposes. The gray superimposed on each colored line depicts the 95% confidence interval in each sex. The quadratic model demonstrated significantly better fit than the linear model for salience network segregation across adulthood (AIC difference = 11.11).

The stargazer package (v5.2.2; Hlavac, 2018) was used to create Tables 2–7. The ggplot2 package was used to create linear and quadratic plots (Figures 1–5; v3.3.3; Wickham, 2016). All figures were created using a colorblind friendly palette *via* RColorBrewer (Neuwirth and Neuwirth, 2023).

**TABLE 2:** This table presents results from linear regressions for estradiol and network segregation in females.

Female estradiol linear associations with network segregation						
	Estradiol β coefficient	Raw *P*-value	*R* ^2^	Adjusted *R*^2^	Residual std. error (df = 55)	F statistic (df = 1; 55)
Au	-0.008	0.738	0.002	-0.016	0.103	0.114
CBBG	0.039	0.313	0.019	0.001	0.157	1.037
COTC	0.914	0.385	0.014	-0.004	4.27	0.769
DA	-0.023	0.392	0.013	-0.005	0.109	0.746
DM	0.018	0.625	0.004	-0.014	0.146	0.242
FPTC	0.015	0.646	0.004	-0.014	0.129	0.214
Sa	0.038	0.259	0.023	0.005	0.138	1.306
SSH	-0.008	0.761	0.002	-0.016	0.111	0.094
SSM	-0.024	0.205	0.029	0.011	0.077	1.646
Vi	-0.013	0.705	0.003	-0.015	0.137	0.146
VA	-0.022	0.731	0.002	-0.016	0.26	0.120

Raw *p*-values are listed, and FDR correction was only performed if raw *p*-value was < 0.05.

**TABLE 3:** This table presents results from linear regressions for progesterone and network segregation in females.

Female progesterone linear associations with network segregation							
	Progesterone β coefficient	Raw *P*-value	FDR corrected *P*-value	*R* ^2^	Adjusted *R*^2^	Residual std. error (df = 59)	F statistic (df = 1; 59)
Au	0.0001	0.575	0.926	0.005	-0.011	0.107	0.318
CBBG	0.0003	0.176	0.484	0.031	0.014	0.166	1.880
COTC	0.015	0.010	0.110	0.109	0.094	3.873	7.247
DA	0.0001	0.674	0.926	0.003	-0.014	0.107	0.179
DM	0.0002	0.255	0.561	0.022	0.005	0.143	1.323
FPTC	0.0004	0.049	0.270	0.064	0.049	0.123	4.067
Sa	0.0003	0.102	0.374	0.045	0.029	0.134	2.764
SSH	0.0001	0.711	0.926	0.002	-0.015	0.116	0.139
SSM	0.00001	0.926	0.926	0.0002	-0.017	0.082	0.009
Vi	0.00004	0.850	0.926	0.001	-0.016	0.133	0.036
VA	-0.0001	0.881	0.926	0.0004	-0.017	0.246	0.023

Raw *p*-values and FDR corrected *p*-values are included. There were no significant findings after FDR correction.

**TABLE 4:** This table presents results from linear regressions for testosterone and network segregation in females.

Female testosterone linear associations with network segregation						
	Testosterone β coefficient	Raw *P*-value	*R* ^2^	Adjusted *R*^2^	Residual std. error (df = 60)	F statistic (df = 1; 60)
Au	-0.0001	0.881	0.0004	-0.016	0.106	0.023
CBBG	0.0003	0.668	0.003	-0.014	0.167	0.187
COTC	0.009	0.561	0.006	-0.011	4.115	0.342
DA	-0.0002	0.715	0.002	-0.014	0.106	0.135
DM	0.001	0.112	0.042	0.026	0.141	2.605
FPTC	0.0001	0.900	0.0003	-0.016	0.126	0.016
Sa	0.001	0.262	0.021	0.005	0.136	1.284
SSH	-0.0001	0.872	0.0004	-0.016	0.116	0.027
SSM	-0.0003	0.283	0.019	0.003	0.08	1.175
Vi	-0.0002	0.676	0.003	-0.014	0.131	0.177
VA	-0.001	0.364	0.014	-0.003	0.245	0.837

Raw *p*-values are listed, and FDR correction was only performed if raw *p*-value was < 0.05.

**TABLE 5:** This table exhibits results from linear regressions for hormone interactions and network segregation in females.

Female hormone level interactions with network segregation											
	Estradiol by progesterone (E*P) β coefficient	Raw E*P *P*-value	Estradiol by testosterone (E*T) β coefficient	Raw E*T *P*-value	Progesterone by testosterone (P*T) β coefficient	Raw P*T *P*-value	FDR corrected *P*-value for estradiol by testosterone (E*T)	*R* ^2^	Adjusted *R*^2^	Residual std. error (df = 48)	F statistic (df = 6; 48)
Au	0.0003	0.669	-0.003	0.072	0.00001	0.360	0.264	0.096	-0.017	0.103	0.851
CBBG	0.001	0.131	-0.003	0.167	0.00001	0.760	0.359	0.133	0.024	0.155	1.225
COTC	-0.029	0.245	-0.056	0.315	0.001	0.111	0.429	0.182	0.079	4.087	1.776
DA	-0.0005	0.489	-0.001	0.511	0.00001	0.507	0.511	0.05	-0.069	0.111	0.422
DM	0.0002	0.792	-0.002	0.333	0	0.856	0.429	0.085	-0.029	0.148	0.747
FPTC	-0.0002	0.786	-0.002	0.196	0.00001	0.496	0.359	0.135	0.027	0.126	1.248
Sa	0.0001	0.940	-0.002	0.359	0.00002	0.363	0.429	0.072	-0.044	0.14	0.619
SSH	0.0002	0.755	-0.003	0.035	0.00003	0.088	0.193	0.133	0.025	0.111	1.23
SSM	0.0001	0.766	-0.002	0.028	0.00002	0.137	0.193	0.157	0.051	0.076	1.485
Vi	-0.001	0.125	0.002	0.390	0.00001	0.711	0.429	0.059	-0.059	0.142	0.497
VA	0.002	0.307	-0.005	0.127	0.00001	0.691	0.349	0.072	-0.044	0.26	0.617

Raw *p*-values and FDR corrected *p*-values are included. FDR correction was only performed if raw *p*-value was < 0.05. There were no significant findings after FDR correction.

**TABLE 6:** This table exhibits results from linear regressions for age and network segregation in females.

Female linear age associations with network segregation							
	Age β coefficient	Raw *P*-value	FDR corrected *P*-value	*R* ^2^	Adjusted *R*^2^	Residual std. error (df = 64)	F statistic (df = 1; 64)
Au	−0.002	0.024	0.088	0.078	0.064	0.105	5.414
CBBG	−0.005	0.01	0.077	0.099	0.085	0.168	7.061
COTC	−0.082	0.044	0.103	0.062	0.047	3.918	4.231
DA	−0.001	0.311	0.342	0.016	0.001	0.106	1.043
DM	−0.002	0.18	0.283	0.028	0.013	0.146	1.843
FPTC	−0.003	0.047	0.103	0.06	0.046	0.126	4.114
Sa	−0.004	0.014	0.077	0.092	0.078	0.141	6.466
SSH	−0.001	0.276	0.337	0.019	0.003	0.113	1.210
SSM	−0.001	0.065	0.119	0.052	0.037	0.077	3.529
Vi	−0.001	0.357	0.357	0.013	-0.002	0.13	0.862
VA	−0.003	0.252	0.337	0.021	0.005	0.241	1.341

Raw *p*-values and FDR corrected *p*-values are included. There were no significant findings after FDR correction.

**TABLE 7:** This table exhibits results from quadratic regressions for age and network segregation in females.

Female quadratic age associations with network segregation							
	Quadratic β coefficient	Raw *P*-value	FDR corrected *P*-value	*R* ^2^	Adjusted *R*^2^	Residual std. error (df = 63)	F statistic (df = 2; 63)
Au	−0.0002	0.014	0.051	0.164	0.138	0.1	6.202
CBBG	−0.0003	0.002	0.011*	0.241	0.217	0.155	10.020
COTC	−0.002	0.391	0.430	0.073	0.044	3.925	2.482
DA	−0.0001	0.071	0.112	0.066	0.036	0.104	2.23
DM	−0.0002	0.043	0.095	0.09	0.061	0.142	3.109
FPTC	−0.0002	0.058	0.106	0.113	0.085	0.124	4.011
Sa	−0.0003	0.0005	0.011*	0.255	0.232	0.129	10.806
SSH	−0.0002	0.035	0.095	0.086	0.057	0.11	2.977
SSM	−0.0001	0.16	0.210	0.082	0.053	0.077	2.808
Vi	−0.0001	0.172	0.210	0.042	0.012	0.129	1.393
VA	−0.0001	0.578	0.578	0.025	-0.006	0.242	0.820

Raw *p*-values and FDR corrected *p*-values are included. Asterisks indicate significance at *p* < 0.05* for FDR corrected values. Only FDR corrected values are interpreted as significant.

## 3. Results

### 3.1. Hormone levels by sex

An ANOVA revealed significant sex differences in testosterone when accounting for age [*F*(1,111) = 79.496, *p* < 0.001; Figure 1], that is testosterone levels were significantly lower in females. ANOVAs did not reveal significant sex differences in estradiol or progesterone levels [*F*(1,105) = 3.318, *p* = 0.071, η^2^ = 0.031], and [*F*(1,109) = 3.270, *p* = 0.073, η^2^ = 0.029], respectively Figure 2.

### 3.2. Network segregation and hormone levels in females

When examining females alone, network segregation was not significantly associated with progesterone, estradiol, or testosterone in females after FDR correction (see Tables 2–4). Similar exploratory analyses were conducted with hormone levels (i.e., estradiol, progesterone, and testosterone; respectively) across participants and in males and all participants (see Supplementary Tables 1–3).

### 3.3. Interactions between hormone levels and network segregation in females

Regressions evaluating combined effects (interactions) of hormone levels (i.e., Estradiol*Progesterone, Estradiol*Testosterone, and Progesterone*Testosterone) as the predictor and network segregation as the outcome were not significant (see Table 5). Similar analyses were conducted in males and all participants (see Supplementary Tables 12, 13). Notably, though the 17β-estradiol*testosterone interaction was associated with segregation in both the sensory somatomotor hand and mouth networks, these did not survive FDR correction.

### 3.4. Linear and quadratic associations between age and network segregation

Network segregation was evaluated in females across the adult lifespan with respect to age. Linear regressions with age as the predictor and network segregation as the outcome revealed no significant associations after corrections for multiple comparisons (see Table 6).

However, in females, regressions with quadratic age as the predictor and network segregation as the outcome, demonstrated significant associations in the cerebellar-basal ganglia (CBBG) [*F*(2, 63) = 10.020, raw *p* = 0.002, FDR adjusted *p* = 0.011] and salience (Sa) [*F*(2, 63) = 10.806, raw *p* < 0.001, FDR adjusted *p* = 0.011] networks (Figures 3, 4 and Table 7). There were no additional significant associations in network segregation and quadratic age for females or males as revealed in our exploratory analyses (detailed results are presented in Table 7).

Comparisons of model fit between linear and quadratic regressions revealed that salience network segregation fits significantly better with a quadratic model as compared to linear in females (AIC difference = 11.11, Figure 3). However, all other comparisons of model fit between linear, and quadratic were not statistically significant (see Supplementary Table 11).

## 4. Discussion

This study investigated network segregation in aging females in the context of sex steroid hormones. Primarily, we were interested in network segregation in adult females, as sex steroid hormone levels—which undergo dramatic changes in females during mid and later life—may impact brain network properties. Understanding differences in network segregation in females in the context of aging and hormone levels stands to provide a greater understanding around factors contributing to functional differences in aging, particularly given that older females are at greater risk for negative outcomes in later life (Gao et al., 1998; Burger, 2008; Lahousse et al., 2014; Alzheimer’s Association, 2022). Somewhat surprisingly, we found no significant relationships between sex steroid hormone levels and network segregation levels in adult females. To our knowledge, this is the first study to directly investigate endogenous hormone levels with network segregation and patterns of aging.

In the context of both endogenous hormone levels and exogenous sex hormone treatments, sex hormones have displayed impacts on brain structure and function (e.g., cortical connectivity, subcortical connectivity, and within-network coherence) (Peper et al., 2011; Taylor et al., 2019; Pritschet et al., 2020). Given the notable vacillations in hormone levels during distinct reproductive stages (Diedrichsen, 2006) and cognitive inefficiencies associated with certain reproductive stages (Greendale et al., 2011; Epperson et al., 2013; Weber et al., 2014; Rentz et al., 2017; Taylor et al., 2019; Pritschet et al., 2020), we expected to find associations between network segregation and hormone levels. However, females did not exhibit significant associations between progesterone, 17β-estradiol, or testosterone and network segregation. These findings are inconsistent with a recent study of network segregation from our group (Ballard et al., 2022c) which demonstrated some differences with reproductive stage, suggesting hormones may play impact network segregation. That is, female reproductive aging is associated with declines in 17β-estradiol and progesterone (Burger, 2008; Harlow et al., 2012; Sundström Poromaa and Gingnell, 2014) and Ballard et al. (2022c) demonstrated significant differences between distinct reproductive stages and network segregation (i.e., COTC, DMN, DA, FPTC, and Sa) in females (Ballard et al., 2022c). Of note, Ballard et al.’s (2022c) findings may be driven by age as they also found linear relationships between age and network segregation in the aforementioned networks. Pritschet et al. (2020) demonstrated that estradiol is associated with increasing global efficiency in the DMN and DA networks, whereas progesterone was associated with reduced coherence throughout the brain (Pritschet et al., 2020). While Pritschet et al.’s (2020) findings suggested influences of estradiol and progesterone on network dynamics, it is difficult to directly compare their findings to ours as their study was based on dense sampling in a young female across a menstrual cycle and evaluated a different aspect of network function. However, it broadly demonstrates the purported relationship between brain network organization and sex steroid hormones in the female brain. Although we cannot directly compare our findings with this and other studies, network segregation is a proxy for measuring functional organization in the brain and may loosely be interpreted as such.

Lastly, the combined impact (interaction) of hormone levels (i.e., estradiol, progesterone, and testosterone) on network segregation did not reveal a relationship with network segregation in females. We found interactions between estradiol and testosterone in SSH and SSM networks for raw *p*-values, but these findings did not survive FDR correction. However, this finding agrees with the study by Moffat (2005), in which they note that testosterone can interact not only with androgen receptors, but also with estradiol receptors, and therefore, its administration can, in some cases, parallel the effects of estradiol on the entire nervous system. An important element in understanding the effects of testosterone on the nervous system is that many of its behavioral and anatomical effects occur after it has been converted to its metabolically active derivatives–estradiol or dihydrotestosterone (Moffat, 2005).

One consideration beyond the scope of the current study is how stages within a female menstrual cycle or menopausal stage could impact brain connectivity. Syan et al. (2017) examined endogenous estradiol, progesterone, and a neuroactive metabolite of progesterone (allopregnanolone) at different menstrual phases and demonstrated an impact of hormone levels in the late luteal phase on resting state connectivity in both cortical and subcortical regions for reproductive aged females (Syan et al., 2017). As mentioned earlier, Pritschet et al. (2020) exhibited an impact of sex hormones on network architecture in tandem with the cycle of a reproductive aged female. Thus, sex steroid hormones have shown a relationship with brain connectivity as it relates to regular menstrual cycle fluctuation. Individual variation exists both in a regular menstrual cycle and menopausal stages (Fehring et al., 2006; Harlow et al., 2012; Boker et al., 2014). Notably, hormone levels are not the sole indicator for identifying distinct reproductive stages. Seminal work in categorizing stages in reproductive aging described principal criteria for determining reproductive stage by changes in cycle regularity and days to years since last cycle (Harlow et al., 2012). Endocrine information such as follicle stimulating hormones, antimullerian hormone, and inhibin-B are used to support categorization by changes in cycle (Harlow et al., 2012). We did not examine menstrual cycle, the above specified endocrine information, menopausal stage, or within individual hormone variance in this investigation. As such, the nature of our analyses and scope of our study may not capture the complexity of female network segregation.

In the context of aging, females did display a significant quadratic relationship between age and network segregation for the CBBG and Sa networks. In both cases, segregation was still increasing through adulthood and highest in midlife with a downturn thereafter. Patterns of connectivity between the cerebellum and basal ganglia have been positively linked to cognitive performance and self-reported balance confidence in older adults (Power et al., 2011; Bernard and Seidler, 2013). Further, in their review Diedrichsen et al. (2009) (Diedrichsen, 2006) implicated the relationship between the cerebellum and basal ganglia as critical for modulating cortical functions such as cognition and relying on subcortical processes. We can see in Figure 3, that both females and males demonstrate an inverted “U-shaped” decline in CBBG network segregation across the span of aging adults. Functionally this pattern in females may be related to the drop in cognition and decreased balance that females experience above and beyond males in aging (Burger, 2008; Pritschet et al., 2020), though we should note that this is speculative as we did not include any behavioral analyses here.

The quadratic age relationship with Sa network segregation in females was somewhat consistent with work from Chan et al. (2014), where they showed a significant quadratic age relationship across female and male participants in “association systems” which included the Sa network, but was aggregated with DMN, FPTC, VA, COTC, and DA (Chan et al., 2014). The Sa network has been associated with “conscious integration of autonomic feedback and responses with internal goals and environmental demands” (Weis et al., 2008). This network has also been conceptualized as an “integral hub” for facilitating communication between the DMN and central executive networks in a triple network model (Van Goozen et al., 1995). Notably, dysfunction in the Sa network has been linked to reduced cognitive performance in older adults (Dimech et al., 2019) and increased connectivity in this network has been linked to Alzheimer’s disease (Morrison et al., 2006). The quadratic relationship seen in females, but not males (see Figure 3), could similarly be highlighting cognitive inefficiencies that have been demonstrated during advanced aging in females (Pritschet et al., 2020).

It is important to emphasize that we did not find significant quadratic age relationships in any additional networks for females, nor any networks in males in our exploratory analyses. These findings are consistent with Lee et al. (2016) study that examined rs-fMRI graph network analysis in the DMN, Sa, and Central Executive network and a quadratic age relationship in both groups of “good” and “poor” cognitive performers (Lee et al., 2016). While Lee et al. (2016) assessed components of both network segregation and network integration (i.e., global efficiency, local efficiency, betweenness centrality, connectivity strength, and nodal degree), their analyses notably varied from ours in that they controlled for gender (Lee et al., 2016). That is, the relationship between quadratic age and segregation in certain networks may be driven by inherent hormonal differences between sexes, but as gender was controlled for in their study that relationship was left unexplored (Lee et al., 2016). Thus, the methodological differences may explain the differences in outcomes relative to what we report here.

Comparisons of model fit demonstrated the quadratic model as a substantially better fit for Sa network segregation and age in females; however, no additional quadratic age models displayed a significantly better fit in our analyses. To our knowledge, associations between quadratic age and network segregation have not been otherwise evaluated or reported. We would suggest that this may be a useful area of investigation in future work. We did not, however, find linear relationships between age and network segregation when examining females or males after FDR correction. Similarly, Grady et al. (2016), did not see linear relationships with age and network segregation, although their examination was specific to the DMN, DA, and FPTC networks (Grady et al., 2016). As stated, earlier Chan et al. (2014) found a significant quadratic age relationship in association systems (Chan et al., 2014); however, these systems were also significantly linearly associated with age. The same study also revealed linear age relationships with network segregation in “sensory-motor systems” which aggregated Hand somato-motor, Visual, Mouth somato-motor, and Auditory within-network segregations (Hausmann, 2005). While we did not replicate Chan et al.’ (2014) findings, we also did not aggregate networks for analysis in the same fashion. Further there are also differences in the data used for analyses with respect to both sample size and length of scans. That is, their sample size was twice as large as our study and scan length was about 5 min per participant. Of note, our approach to data collection (guided by recent scientific advancement and literature) often produces more reliable data (Pannunzi et al., 2017) as we collected 24 total minutes of resting state scans, alternating from anterior to posterior slice collection. Thus, Chan et al.’ (2014) investigation of network segregation in sensory-motor systems may not be appropriate for drawing direct comparisons to our data given methodological advancements in recent years resulting in differences in data collection parameters. Additional studies evaluating linear and quadratic relationships by sex are warranted, particularly with larger samples that include longer resting state acquisition times such as that used here.

Overall, we found quadratic age relationships for females in CBBG and Sa network segregation. We did not find significant impacts of individual or combined hormone levels on within-network segregation in the networks of interest. Future research would benefit from examining inter-individual differences over time to gain more insight into subtle influences of endogenous hormones. Another relevant area of research would be examining individual differences of network segregation over time in those taking hormonal contraceptives or receiving hormone replacement therapy to better understand the dynamic interplay between sex steroid hormones and brain network organization.

### 4.1. Limitations

There are several limitations relevant to the present investigation. Namely, the variability and collection of hormone assays and the cross-sectional nature of these data. It is important to recognize that hormone levels can vary almost as much within a naturally cycling female as they can between naturally cycling females (Fehring et al., 2006; Bernard and Seidler, 2013). Furthermore, the hormone assay was collected on a different day than the scan as freezing the sample at a particular temperature is required immediately, and our study design incorporated a delay to allow for additional measures of activity and questionnaire completion (not related to the research questions presented here). The large variance of hormone levels within a cycle implies that levels could change in a matter of days for reproductive age females. Thus, given the age range of our female sample, the offset between the hormone sample and the scan may impact these relationships for those that still have regular menstrual cycles. While this is only a small subset of this sample, it is an important limitation, nonetheless. Hormone levels vary within and between naturally cycling females so examining sex hormone levels in the same participant over time would account for some individual variance in hormone fluctuations. Regarding menopause, our study did ask about participant start date of menopause (date of final menstrual period) with additional questions to help categorize menopausal stages. That is, all participants completed 6-month menstrual diaries after undergoing the brain imaging session. The impact of menopausal stage is a question of interest for us, and indeed we have studied this recently (Ballard et al., 2022b). However, sex hormone levels have been directly associated with menopausal stages (Burger et al., 2007) and we are interested in the variability in hormone levels more generally, as this also provides a quantitative measure, as opposed to self-report menstrual cycle information. Regarding obstetrics history, we do not have that information for this sample, although the literature suggests that parity has been positively related to brain volume in aging females (de Lange et al., 2019). Several females who had undergone hysterectomy were included in the study, but the surgeries were dated over 10 years prior to enrollment in the study. As such, it was assumed that hormones had reached a stable low. Another notable limitation is our sex hormone analyses did not account for age. This was intentional as preliminary analyses demonstrated age was significantly correlated with female estradiol and progesterone (*p* < 0.01) and testosterone maintains a relatively consistent level throughout for mid- to older- age females (Andersen et al., 2011). Thus, incorporating age into hormone-based analyses would account for very similar variance to sex hormone levels. Sleep patterns have also demonstrated a relationship with sex hormone levels (Li et al., 2015; Brown and Gervais, 2022). Indeed, we do find this aspect important to investigate and it has been explored by proxy with this sample in the context of reproductive stages (Ballard et al., 2022b). Lastly, our sample size was relatively small in relation to the number of analyses conducted. With this in mind, we were very targeted in our analyses and applied a correction for multiple comparisons (FDR correction). However, replication of this study in a larger sample is warranted.

## 5. Conclusion

This study demonstrated a quadratic relationship between aging and network segregation for the CBBG and Sa networks in females. In both cases, segregation was still increasing through adulthood and highest in midlife with a downturn thereafter. These networks are functionally related to cognitive performance, balance, and integrating autonomic feedback in response to environmental demands (Bernard and Seidler, 2013; Chan et al., 2014; Hausman et al., 2020).

Prior research has shown that the sex hormones can regulate neurogenesis, inflammatory processes, impact network segregation, and may also play a role in regulating cognitive and affective processes, mainly in the aging process (Syan et al., 2017; Foo et al., 2021). Furthermore, the notable variability in hormone levels in certain reproductive stages and cognitive deficits associated with specific reproductive stages did not have an impact on network segregation as was expected (Greendale et al., 2011; Epperson et al., 2013; Weber et al., 2014; Rentz et al., 2017; Taylor et al., 2019; Pritschet et al., 2020).

Future studies could focus on examining participants longitudinally and pairing these types of data with behavioral outcomes.

## Data availability statement

The raw data supporting the conclusions of this article will be made available by the authors, without undue reservation.

## Ethics statement

The studies involving human participants were reviewed and approved by the Texas A&M Institutional Review Board. The patients/participants provided their written informed consent to participate in this study.

## Author contributions

TH, HB, TJ, and SC completed the data collection. TH, TM, HB, and TJ conducted the data analyses under the guidance and supervision of JAB. TH and TM drafted the manuscript with input from all authors and again under the guidance of JAB. All authors contributed to the conceptualization of this project.

## Footnotes

1
https://salimetrics.com/saliva-collection-training-videos/

2
https://salimetrics.com/analyte/salivary-estradiol/

3
https://github.com/jmtyszka/bidskit

4
https://fmriprep.org/

## References

[B1] Aggarwal N. Patel H. Mehta L. Sanghani R. Lundberg G. Lewis S. (2018). Sex differences in ischemic heart disease: Advances, obstacles, and next steps. *Circ. Cardiovasc. Qual. Outcomes* 11:e004437. 10.1161/CIRCOUTCOMES.117.004437 29449443

[B2] Allen E. Erhardt E. Damaraju E. Gruner W. Segall J. Silva R. (2011). A baseline for the multivariate comparison of resting-state networks. *Front. Syst. Neurosci.* 5:2. 10.3389/fnsys.2011.00002 21442040 PMC3051178

[B3] Alzheimer’s Association. (2022). *Alzheimer’s disease facts and figures.* Chicago, IL: Alzheimer’s Association. 10.1002/alz.12638 35289055

[B4] Andersen M. Alvarenga T. Mazaro-Costa R. Hachul H. Tufik S. (2011). The association of testosterone, sleep, and sexual function in men and women. *Brain Res.* 1416 80–104. 10.1016/j.brainres.2011.07.060 21890115

[B5] Ballard H. Jackson T. B. Hicks T. Cox S. Miller A. Maldonado T. (2022a). Hormone-sleep interactions predict cerebellar connectivity and behavior in aging females. *bioRxiv [Preprint]* 10.1101/2022.08.30.505858 PMC10149037 36709633

[B6] Ballard H. Jackson T. Miller A. Hicks T. Bernard J. (2022c). Age-related differences in functional network segregation in the context of sex and reproductive stage. *Hum. Brain Mapp.* 10.1101/2022.03.28.486067 [Epub ahead of print]. PMC9980887 36541480

[B7] Ballard H. K. Jackson T. B. Hicks T. H. Bernard J. A. (2022b). The association of reproductive stage with lobular cerebellar network connectivity across female adulthood. *Neurobiol. Aging* 117 139–150. 10.1016/j.neurobiolaging.2022.05.014 35738086 PMC10149146

[B8] Bernard J. Seidler R. (2013). Relationships between regional cerebellar volume and sensorimotor and cognitive function in young and older adults. *Cerebellum* 12 721–737. 10.1007/s12311-013-0481-z 23625382 PMC3820158

[B9] Bernard J. Leopold D. Calhoun V. Mittal V. (2015). Regional cerebellar volume and cognitive function from adolescence to late middle age. *Hum. Brain Mapp.* 36 1102–1120. 10.1002/hbm.22690 25395058 PMC4323630

[B10] Bixo M. Andersson A. Winblad B. Purdy R. Bäckström T. (1997). Progesterone, 5alpha-pregnane-3,20-dione and 3alpha-hydroxy-5alpha-pregnane-20-one in specific regions of the human female brain in different endocrine states. *Brain Res.* 764 173–178. 10.1016/S0006-8993(97)00455-1 9295207

[B11] Bohon C. Welch H. (2021). Quadratic relations of BMI with depression and brain volume in children: Analysis of data from the ABCD study. *J. Psychiatr. Res.* 136 421–427. 10.1016/j.jpsychires.2021.02.038 33657461 PMC8225589

[B12] Boker S. Neale M. Klump K. (2014). “A differential equations model for the ovarian hormone cycle,” in *Handbook of developmental systems theory and methodology*, eds Molenaar P. C. M. Lerner R. M. Newell K. M. (New York, NY: The Guilford Press), 369–394.

[B13] Brown A. Gervais N. (2022). Role of ovarian hormones in the modulation of sleep in females across the adult lifespan. Endocrinology. 2020 Sep 1;161(9): bqaa128. Erratum in. *Endocrinology* 163:bqab227. 10.1210/endocr/bqaa128 32735650 PMC7450669

[B14] Burger H. (2008). The menopausal transition–endocrinology. *J. Sex. Med.* 5 2266–2273. 10.1111/j.1743-6109.2008.00921.x 18624962

[B15] Burger H. G. Hale G. E. Robertson D. M. Dennerstein L. (2007). A review of hormonal changes during the menopausal transition: Focus on findings from the Melbourne Women’s Midlife Health Project. *Hum. Reprod. Update* 13 559–565. 10.1093/humupd/dmm020 17630397

[B16] Burnham K. Anderson D. (2004). Multimodel inference: Understanding AIC and BIC in model selection. *Sociol. Methods Res.* 33 261–304. 10.1177/0049124104268644

[B17] Cassady K. Gagnon H. Lalwani P. Simmonite M. Foerster B. Park D. (2019). Sensorimotor network segregation declines with age and is linked to GABA and to sensorimotor performance. *Neuroimage* 186 234–244. 10.1016/j.neuroimage.2018.11.008 30414983 PMC6338503

[B18] Chan M. Park D. Savalia N. Petersen S. Wig G. (2014). Decreased segregation of brain systems across the healthy adult lifespan. *Proc. Natl Acad. Sci. U. S. A.* 111 E4997–E5006. 10.1073/pnas.1415122111 25368199 PMC4246293

[B19] Cosgrove K. Mazure C. Staley J. (2007). Evolving knowledge of sex differences in brain structure, function, and chemistry. *Biol. Psychiatry* 62 847–855. 10.1016/j.biopsych.2007.03.001 17544382 PMC2711771

[B20] de Lange A. M. G. Kaufmann T. van der Meer D. Maglanoc L. A. Alnæs D. Moberget T. (2019). Population-based neuroimaging reveals traces of childbirth in the maternal brain. *Proc. Natl. Acad. Acad. Sci. U.S.A.* 116 22341–22346. 10.1073/pnas.1910666116 31615888 PMC6825266

[B21] Di Martino A. Scheres A. Margulies D. Kelly A. Uddin L. Shehzad Z. (2008). Functional connectivity of human striatum: A resting state FMRI study. *Cereb. Cortex* 18 2735–2747. 10.1093/cercor/bhn041 18400794

[B22] Diamond M. (1991). Hormonal effects on the development or cerebral lateralization. *Psychoneuroendocrinology* 16 121–129. 10.1016/0306-4530(91)90074-4 1961835

[B23] Diedrichsen J. (2006). A spatially unbiased atlas template of the human cerebellum. *Neuroimage* 33 127–138. 10.1016/j.neuroimage.2006.05.056 16904911

[B24] Diedrichsen J. Balsters J. Flavell J. Cussans E. Ramnani N. (2009). A probabilistic MR atlas of the human cerebellum. *Neuroimage* 46 39–46. 10.1016/j.neuroimage.2009.01.045 19457380

[B25] Dimech C. Anderson J. Lockrow A. Spreng R. Turner G. (2019). Sex differences in the relationship between cardiorespiratory fitness and brain function in older adulthood. *J Appl Physiol.* 126 1032–1041. 10.1152/japplphysiol.01046.2018 30702974 PMC6485686

[B26] Epperson C. Sammel M. Freeman E. (2013). Menopause effects on verbal memory: Findings from a longitudinal community cohort. *J. Clin. Endocrinol. Metab.* 98 3829–3838. 10.1210/jc.2013-1808 23836935 PMC3763981

[B27] Fehring R. Schneider M. Raviele K. (2006). Variability in the phases of the menstrual cycle. *J Obstetr. Gynecol. Neonatal Nurs.* 35 376–384. 10.1111/j.1552-6909.2006.00051.x 16700687

[B28] Filippi M. Valsasina P. Misci P. Falini A. Comi G. Rocca M. (2013). The organization of intrinsic brain activity differs between genders: A resting-state fMRI study in a large cohort of young healthy subjects. *Hum. Brain Mapp.* 34 1330–1343. 10.1002/hbm.21514 22359372 PMC6870508

[B29] Foo H. Thalamuthu A. Jiang J. Koch F. Mather K. Wen W. (2021). Age- and sex-related topological organization of human brain functional networks and their relationship to cognition. *Front. Aging Neurosci.* 13:758817. 10.3389/fnagi.2021.758817 34975453 PMC8718995

[B30] Gao S. Hendrie H. Hall K. Hui S. (1998). The relationships between age, sex, and the incidence of dementia and Alzheimer disease: A meta-analysis. *Arch. Gen. Psychiatry* 55 809–815. 10.1001/archpsyc.55.9.809 9736007

[B31] Goldstone A. Mayhew S. Przezdzik I. Wilson R. Hale J. Bagshaw A. (2016). Gender specific re-organization of resting-state networks in older age. *Front. Aging Neurosci.* 8:285. 10.3389/fnagi.2016.00285 27932978 PMC5122714

[B32] Grady C. Sarraf S. Saverino C. Campbell K. (2016). Age differences in the functional interactions among the default, frontoparietal control, and dorsal attention networks. *Neurobiol. Aging* 41 159–172. 10.1016/j.neurobiolaging.2016.02.020 27103529

[B33] Greendale G. Derby C. Maki P. (2011). Perimenopause, and cognition. *Obstetr. Gynecol. Clin. North Am.* 38 519–535. 10.1016/j.ogc.2011.05.007 21961718 PMC3185244

[B34] Harlow S. Gass M. Hall J. Lobo R. Maki P. Rebar R. (2012). Executive summary of the stages of reproductive aging workshop + 10: Addressing the unfinished agenda of staging reproductive aging. *Fertil. Steril.* 97 1159–1168. 10.1016/j.fertnstert.2012.01.128 22341880 PMC3340904

[B35] Harms M. Somerville L. Ances B. Andersson J. Barch D. Bastiani M. (2018). Extending the human connectome project across ages: Imaging protocols for the lifespan development and aging projects. *Neuroimage.* 183 972–984. 10.1016/j.neuroimage.2018.09.060 30261308 PMC6484842

[B36] Hausman H. Jackson T. Goen J. Bernard J. (2020). From synchrony to asynchrony: Cerebellar-basal ganglia functional circuits in young and older adults. *Cereb. Cortex* 30 718–729. 10.1093/cercor/bhz121 31219563

[B37] Hausmann M. (2005). Hemispheric asymmetry in spatial attention across the menstrual cycle. *Neuropsychologia* 43 1559–1567. 10.1016/j.neuropsychologia.2005.01.017 16009238

[B38] Hlavac M. (2018). *Stargazer: Well-formatted regression and summary statistics tables. R package version, 5.2.2.*

[B39] Jacobson C. Csernus V. Shryne J. Gorski R. (1981). The influence of gonadectomy, androgen exposure, or a gonadal graft in the neonatal rat on the volume of the sexually dimorphic nucleus of the preoptic area. *J. Neurosci.* 1 1142–1147. 10.1523/JNEUROSCI.01-10-01142.1981 7288477 PMC6564210

[B40] Jenkinson M. Beckmann C. Behrens T. Woolrich M. Smith S. (2012). FSL. *Neuroimage* 62 782–790. 10.1016/j.neuroimage.2011.09.015 21979382

[B41] Lahousse L. Maes B. Ziere G. Loth D. Verlinden V. Zillikens M. (2014). Adverse outcomes of frailty in the elderly: The Rotterdam Study. *Eur. J. Epidemiol.* 29 419–427. 10.1007/s10654-014-9924-1 24935872

[B42] Lee A. Tan M. Qiu A. (2016). Distinct aging effects on functional networks in good and poor cognitive performers. *Front. Aging Neurosci.* 8:215. 10.3389/fnagi.2016.00215 27667972 PMC5016512

[B43] Li D. Romans S. De Souza M. Murray B. Einstein G. (2015). Actigraphic and self-reported sleep quality in women: Associations with ovarian hormones and mood. *Sleep Med.* 16 1217–1224. 10.1016/j.sleep.2015.06.009 26429749

[B44] Lüdecke D. (2022). *sjstats: Statistical functions for regression models.* Genève: Zenodo.

[B45] Moffat S. (2005). Effects of testosterone on cognitive and brain aging in elderly men. *Ann. N. Y. Acad. Sci.* 1005 80–92. 10.1196/annals.1323.014 16387720

[B46] Morrison J. Brinton R. Schmidt P. Gore A. (2006). Estrogen, menopause, and the aging brain: How basic neuroscience can inform hormone therapy in women. *J. Neurosci.* 26 10332–10348. 10.1523/JNEUROSCI.3369-06.2006 17035515 PMC6674699

[B47] Neuwirth E. Neuwirth M. (2023). *The apache software foundation, licensed under the apache license, version 2.0*. Available online at: https://www.apache.org/licenses/LICENSE-2.0 (accessed December 15, 2022).

[B48] Ogle D. Doll J. Wheeler P. Dinno A. (2022). *FSA: Fisheries stock analysis.* Available online at: https://fishr-core-team.github.io/FSA/authors.html (accessed December 15, 2022).

[B49] Osterlund M. Gustafsson J. Keller E. Hurd Y. (2000). Estrogen receptor beta (ERbeta) messenger ribonucleic acid (mRNA) expression within the human forebrain: Distinct distribution pattern to ERalpha mRNA. *J. Clin. Endocrinol. Metab.* 85 3840–3846. 10.1210/jc.85.10.3840 11061547

[B50] Pannunzi M. Hindriks R. Bettinardi R. Wenger E. Lisofsky N. Martensson J. (2017). Resting-state fMRI correlations: From link-wise unreliability to whole brain stability. *Neuroimage* 157 250–262. 10.1016/j.neuroimage.2017.06.006 28599964

[B51] Peper J. van den Heuvel M. Mandl R. Hulshoff Pol H. van Honk J. (2011). Sex steroids and connectivity in the human brain: A review of neuroimaging studies. *Psychoneuroendocrinology* 36 1101–1113. 10.1016/j.psyneuen.2011.05.004 21641727

[B52] Power J. Cohen A. Nelson S. Wig G. Barnes K. Church J. (2011). Functional network organization of the human brain. *Neuron* 72 665–678. 10.1016/j.neuron.2011.09.006 22099467 PMC3222858

[B53] Pritschet L. Santander T. Taylor C. Layher E. Yu S. Miller M. (2020). Functional reorganization of brain networks across the human menstrual cycle. *Neuroimage* 220:117091. 10.1016/j.neuroimage.2020.117091 32621974

[B54] R Core Team. (2021). *The R project for statistical computing.* Vienna: R Foundation for Statistical Computing.

[B55] Raparelli V. Nocella C. Proietti M. Romiti G. Corica B. Bartimoccia S. (2022). Testosterone-to-estradiol ratio and platelet thromboxane release in ischemic heart disease: The EVA project. *J. Endocrinol. Invest.* 45 1367–1377. 10.1007/s40618-022-01771-0 35262860 PMC9184432

[B56] Rentz D. Weiss B. Jacobs E. Cherkerzian S. Klibanski A. Remington A. (2017). Sex differences in episodic memory in early midlife: Impact of reproductive aging. *Menopause* 24 400–408. 10.1097/GME.0000000000000771 27824681 PMC5365356

[B57] Roof R. Havens M. (1992). Testosterone improves maze performance and induces development of a male hippocampus in females. *Brain Res.* 572 310–313. 10.1016/0006-8993(92)90491-Q 1611529

[B58] Sakamoto Y. Ishiguro M. Kitagawa G. (1986). *Akaike information criterion statistics.* Dordrecht: D. Reidel.

[B59] Salimetrics. (2022). *Salivary Estradiol –: @Salimetrics.* Carlsbad, CA: Salimetrics.

[B60] Scheinost D. Finn E. Tokoglu F. Shen X. Papademetris X. Hampson M. (2015). Sex differences in normal age trajectories of functional brain networks. *Hum. Brain Mapp.* 36 1524–1535. 10.1002/hbm.22720 25523617 PMC5522589

[B61] Sundström Poromaa I. Gingnell M. (2014). Menstrual cycle influence on cognitive function and emotion processing-from a reproductive perspective. *Front. Neurosci.* 8:380. 10.3389/fnins.2014.00380 25505380 PMC4241821

[B62] Syan S. Minuzzi L. Costescu D. Smith M. Allega O. Coote M. (2017). Influence of endogenous estradiol, progesterone, allopregnanolone, and dehydroepiandrosterone sulfate on brain resting state functional connectivity across the menstrual cycle. *Fertil. Steril.* 107 1246–1255.e4. 10.1016/j.fertnstert.2017.03.021 28476183

[B63] Taylor C. Pritschet L. Yu S. Jacobs E. (2019). Applying a Women’s health lens to the study of the aging brain. *Front. Hum. Neurosci.* 13:224. 10.3389/fnhum.2019.00224 31333434 PMC6625223

[B64] Toffoletto S. Lanzenberger R. Gingnell M. Sundström-Poromaa I. Comasco E. (2014). Emotional and cognitive functional imaging of estrogen and progesterone effects in the female human brain: A systematic review. *Psychoneuroendocrinology* 50 28–52. 10.1016/j.psyneuen.2014.07.025 25222701

[B65] Van Goozen S. Cohen-Kettenis P. Gooren L. Frijda N. Van de Poll N. (1995). Gender differences in behaviour: Activating effects of cross-sex hormones. *Psychoneuroendocrinology* 20 343–363. 10.1016/0306-4530(94)00076-X 8532819

[B66] van Koeverden I. de Bakker M. Haitjema S. van der Laan S. de Vries J. Hoefer I. (2019). Testosterone to oestradiol ratio reflects systemic and plaque inflammation and predicts future cardiovascular events in men with severe atherosclerosis. *Cardiovasc. Res.* 115 453–462. 10.1093/cvr/cvy188 30052805

[B67] Weber M. Maki P. McDermott M. (2014). Cognition, and mood in perimenopause: A systematic review and meta-analysis. *J. Steroid Biochem. Mol. Biol.* 142 90–98. 10.1016/j.jsbmb.2013.06.001 23770320 PMC3830624

[B68] Weis S. Hausmann M. Stoffers B. Vohn R. Kellermann T. Sturm W. (2008). Estradiol modulates functional brain organization during the menstrual cycle: An analysis of interhemispheric inhibition. *J. Neurosci.* 28 13401–13410. 10.1523/JNEUROSCI.4392-08.2008 19074013 PMC6671753

[B69] Weiser M. Foradori C. Handa R. (2008). Estrogen receptor beta in the brain: From form to function. *Brain Res. Rev.* 57 309–320. 10.1016/j.brainresrev.2007.05.013 17662459 PMC2374745

[B70] Whitfield-Gabrieli S. Nieto-Castanon A. (2012). Conn: A functional connectivity toolbox for correlated and anticorrelated brain networks. *Brain Connect.* 2 125–141. 10.1089/brain.2012.0073 22642651

[B71] Wickham H. (2016). *Ggplot2: Elegant graphics for data analysis.* Midtown Manhattan, NY: Springer International Publishing. 10.1007/978-3-319-24277-4

[B72] Zar Jerrold H. (1996). *Biostatistical analysis*, 3rd Edn. Upper Saddle River: Prentice Hall, 662.

[B73] Zhang C. Cahill N. Arbabshirani M. White T. Baum S. Michael A. (2016). Sex and age effects of functional connectivity in early adulthood. *Brain Connect.* 6 700–713. 10.1089/brain.2016.0429 27527561 PMC5105352

