# Supplementary material for: Network segregation in aging females and evaluation of the impact of sex steroid hormones
Source: Front Hum Neurosci. 2023 Feb 1;17:1059091. doi: 10.3389/fnhum.2023.1059091 (PMC9929548; doi:10.3389/fnhum.2023.1059091)

Supplement material

Supplementary Table 1. This table presents results from linear regressions for estradiol and network segregation in males. Raw *p*-values are listed, and FDR correction was only performed if raw *p* -value was <.05.

Male Estradiol Linear Associations with Network Segregation						
	**Estradiol β coefficient**	**Raw *p*-value**	**R^2**	**Adjusted R^2**	**Residual Std. Error (df = 48)**	**F Statistic (df = 1; 48)**
**Au**	0.011	0.628	0.005	-0.016	0.092	0.239
**CBBG**	-0.01	0.817	0.001	-0.02	0.168	0.054
**COTC**	-0.362	0.466	0.011	-0.009	1.966	0.541
**DA**	-0.008	0.783	0.002	-0.019	0.12	0.077
**DM**	-0.01	0.772	0.002	-0.019	0.136	0.085
**FPTC**	-0.059	0.102	0.055	0.035	0.142	2.789
**Sa**	0.0003	0.994	0	-0.021	0.132	0.0001
**SSH**	-0.006	0.835	0.001	-0.02	0.111	0.044
**SSM**	0.01	0.571	0.007	-0.014	0.067	0.327
**Vi**	0.026	0.342	0.019	-0.002	0.108	0.922
**VA**	0.009	0.832	0.001	-0.02	0.165	0.046

Supplementary Table 2. This table presents results from linear regressions for progesterone and network segregation in males. Raw *p*-values are listed, and FDR correction was only performed if raw *p* -value was <.05.

Male Progesterone Linear Associations with Network Segregation						
	**Progesterone β coefficient**	**Raw *p*-value**	**R^2**	**Adjusted R^2**	**Residual Std. Error (df = 48)**	**F Statistic (df = 1; 48)**
**Au**	0.0004	0.240	0.029	0.008	0.101	1.416
**CBBG**	-0.0002	0.686	0.003	-0.017	0.166	0.166
**COTC**	0.007	0.267	0.026	0.005	1.922	1.264
**DA**	0.00002	0.951	0.0001	-0.021	0.12	0.004
**DM**	0.0002	0.723	0.003	-0.018	0.141	0.128
**FPTC**	-0.0004	0.388	0.016	-0.005	0.146	0.76
**Sa**	0.0001	0.792	0.001	-0.019	0.131	0.071
**SSH**	-0.0001	0.754	0.002	-0.019	0.112	0.099
**SSM**	0.0002	0.381	0.016	-0.004	0.067	0.782
**Vi**	0.0004	0.205	0.033	0.013	0.108	1.656
**VA**	0.00003	0.955	0.0001	-0.021	0.167	0.003

Supplementary Table 3. This table presents results from linear regressions for progesterone and network segregation in males. Raw *p*-values are listed, and FDR correction was only performed if raw *p* -value was <.05.

Male Testosterone Linear Associations with Network Segregation						
	**Testosterone β coefficient**	**Raw *p*-value**	**R^2**	**Adjusted R^2**	**Residual Std. Error (df = 49)**	**F Statistic (df = 1; 49)**
**Au**	0.0002	0.493	0.01	-0.011	0.1	0.478
**CBBG**	-0.0004	0.423	0.013	-0.007	0.165	0.656
**COTC**	0.001	0.867	0.001	-0.02	1.947	0.029
**DA**	-0.0001	0.850	0.001	-0.02	0.12	0.036
**DM**	-0.00001	0.976	0.00002	-0.02	0.141	0.001
**FPTC**	-0.0003	0.473	0.011	-0.01	0.145	0.525
**Sa**	0.0001	0.816	0.001	-0.019	0.13	0.055
**SSH**	-0.0003	0.354	0.018	-0.002	0.11	0.879
**SSM**	0.0001	0.781	0.002	-0.019	0.066	0.078
**Vi**	0.0004	0.253	0.027	0.007	0.106	1.342
**VA**	0.0003	0.517	0.009	-0.012	0.165	0.428

Supplementary Table 4. This table presents results from linear regressions for estradiol and network segregation across male and female participants. Raw *p*-values are listed, and FDR correction was only performed if raw *p* -value was <.05.

Estradiol Linear Associations with Network Segregation						
	**Estradiol β coefficient**	**Raw *p*-value**	**R^2**	**Adjusted R^2**	**Residual Std. Error (df = 105)**	**F Statistic (df = 1; 105)**
**Au**	0.006	0.717	0.001	-0.008	0.098	0.132
**CBBG**	0.017	0.538	0.004	-0.006	0.161	0.384
**COTC**	0.45	0.446	0.006	-0.004	3.42	0.586
**DA**	-0.012	0.557	0.003	-0.006	0.114	0.348
**DM**	0.01	0.688	0.002	-0.008	0.142	0.163
**FPTC**	-0.017	0.472	0.005	-0.005	0.137	0.522
**Sa**	0.028	0.239	0.013	0.004	0.137	1.402
**SSH**	-0.006	0.743	0.001	-0.008	0.11	0.109
**SSM**	-0.007	0.601	0.003	-0.007	0.073	0.276
**Vi**	0.009	0.683	0.002	-0.008	0.124	0.168
**VA**	-0.011	0.776	0.001	-0.009	0.219	0.081

Supplementary Table 5. This table presents results from linear regressions for progesterone and network segregation across male and female participants. Raw *p*-values and FDR corrected *p*-values are included. Asterisks indicate significance at *p*<.05* for FDR corrected values. Only FDR corrected values are interpreted as significant.

Progesterone Linear Associations with Network Segregation							
	**Progesterone β coefficient**	**Raw *p*-value**	**FDR Corrected *p*-value**	**R^2**	**Adjusted R^2**	**Residual Std. Error (df = 109)**	**F Statistic (df = 1; 109)**
**Au**	0.0002	0.187	0.411	0.016	0.007	0.105	1.767
**CBBG**	0.0002	0.245	0.449	0.012	0.003	0.165	1.37
**COTC**	0.014	0.001	0.011*	0.101	0.093	3.142	12.257
**DA**	0.0001	0.563	0.746	0.003	-0.006	0.113	0.337
**DM**	0.0003	0.163	0.411	0.018	0.009	0.142	1.98
**FPTC**	0.0002	0.163	0.411	0.018	0.009	0.135	1.973
**Sa**	0.0003	0.056	0.308	0.033	0.024	0.133	3.756
**SSH**	0.00004	0.799	0.836	0.001	-0.009	0.113	0.066
**SSM**	0.00005	0.610	0.746	0.002	-0.007	0.075	0.263
**Vi**	0.0001	0.435	0.684	0.006	-0.004	0.122	0.615
**VA**	-0.0001	0.836	0.836	0.0004	-0.009	0.212	0.043

Supplementary Table 6. This table presents results from linear regressions for testosterone and network segregation across male and female participants. Raw *p*-values are listed, and FDR correction was only performed if raw *p* -value was <.05.

Testosterone Linear Associations with Network Segregation						
	**Testosterone β coefficient**	**Raw *p*-value**	**R^2**	**Adjusted R^2**	**Residual Std. Error (df = 111)**	**F Statistic (df = 1; 111)**
**Au**	-0.0002	0.288	0.01	0.001	0.105	1.144
**CBBG**	-0.0002	0.433	0.006	-0.003	0.165	0.62
**COTC**	-0.004	0.519	0.004	-0.005	3.331	0.419
**DA**	-0.0002	0.324	0.009	-0.0001	0.112	0.983
**DM**	-0.0001	0.822	0.0005	-0.009	0.143	0.051
**FPTC**	-0.0002	0.305	0.009	0.001	0.134	1.062
**Sa**	-0.0002	0.440	0.005	-0.004	0.135	0.601
**SSH**	-0.0002	0.358	0.008	-0.001	0.112	0.855
**SSM**	-0.0001	0.498	0.004	-0.005	0.074	0.464
**Vi**	-4E-05	0.840	0.0004	-0.009	0.121	0.041
**VA**	0	0.998	0	-0.009	0.212	0.00001

Supplementary Table 7. This table presents results from linear regressions for linear age and network segregation in males. Raw *p*-values are listed, and FDR correction was only performed if raw *p* -value was <.05.

Male Linear Age Associations with Network Segregation						
	**Age β coefficient**	**Raw *p*-value**	**R^2**	**Adjusted R^2**	**Residual Std. Error (df = 53)**	**F Statistic (df = 1; 53)**
**Au**	-0.001	0.389	0.014	-0.005	0.098	0.755
**CBBG**	-0.001	0.538	0.007	-0.012	0.164	0.385
**COTC**	-0.022	0.207	0.03	0.012	1.882	1.635
**DA**	-0.001	0.533	0.007	-0.011	0.117	0.395
**DM**	0.001	0.606	0.005	-0.014	0.14	0.269
**FPTC**	-0.001	0.644	0.004	-0.015	0.143	0.217
**Sa**	-0.001	0.286	0.022	0.003	0.127	1.166
**SSH**	-0.0005	0.638	0.004	-0.015	0.109	0.224
**SSM**	-0.0005	0.452	0.011	-0.008	0.068	0.575
**Vi**	-0.002	0.081	0.057	0.039	0.114	3.184
**VA**	-0.0004	0.796	0.001	-0.018	0.166	0.068

Supplementary Table 8. This table presents results from linear regressions for age and network segregation across male and female participants. Raw *p*-values and FDR corrected *p*-values are included. Asterisks indicate significance at *p*<.05* for FDR corrected values. Only FDR corrected values are interpreted as significant.

Linear Age Associations with Network Segregation							
	**Age β coefficient**	**Raw *p*-value**	**FDR Corrected *p*-value**	**R^2**	**Adjusted R^2**	**Residual Std. Error (df = 117)**	**F Statistic (df = 1; 117)**
**Au**	-0.001	0.074	0.178	0.027	0.019	0.103	3.273
**CBBG**	-0.002	0.106	0.194	0.022	0.014	0.165	2.655
**COTC**	-0.047	0.040	0.178	0.036	0.028	3.214	4.337
**DA**	-0.001	0.325	0.397	0.008	-0.0002	0.111	0.977
**DM**	-0.0002	0.879	0.879	0.0002	-0.008	0.143	0.023
**FPTC**	-0.001	0.212	0.333	0.013	0.005	0.134	1.581
**Sa**	-0.002	0.079	0.178	0.026	0.018	0.133	3.154
**SSH**	-0.001	0.383	0.421	0.007	-0.002	0.111	0.77
**SSM**	-0.001	0.073	0.178	0.027	0.019	0.073	3.282
**Vi**	-0.002	0.081	0.178	0.026	0.018	0.123	3.115
**VA**	-0.002	0.283	0.389	0.01	0.001	0.211	1.168

Supplementary Table 9. This table presents results from quadratic regressions for age and network segregation in male participants. Raw *p*-values and FDR corrected *p*-values are included. Asterisks indicate significance at *p*<.05* for FDR corrected values. Only FDR corrected values are interpreted as significant.

Male Quadratic Age Associations with Network Segregation							
	**Quadratic Age β coefficient**	**Raw *p*-value**	**FDR Corrected *p*-value**	**R^2**	**Adjusted R^2**	**Residual Std. Error (df = 52)**	**F Statistic (df = 2; 52)**
**Au**	-0.0001	0.084	0.462	0.07	0.034	0.096	1.954
**CBBG**	-0.0003	0.020	0.220	0.107	0.073	0.157	3.116
**COTC**	-0.0002	0.904	0.962	0.03	-0.007	1.899	0.81
**DA**	0.00001	0.941	0.962	0.008	-0.031	0.119	0.197
**DM**	-0.0001	0.409	0.962	0.018	-0.02	0.141	0.481
**FPTC**	0.0001	0.495	0.962	0.013	-0.025	0.143	0.344
**Sa**	-0.00001	0.922	0.962	0.022	-0.016	0.128	0.577
**SSH**	-0.00003	0.731	0.962	0.007	-0.032	0.11	0.17
**SSM**	0.00001	0.756	0.962	0.013	-0.025	0.068	0.332
**Vi**	0	0.962	0.962	0.057	0.02	0.115	1.563
**VA**	-0.00004	0.713	0.962	0.004	-0.034	0.167	0.102

Supplementary Table 10. This table presents results from quadratic regressions for age and network segregation across male and female participants. Raw *p*-values and FDR corrected *p*-values are included. Asterisks indicate significance at *p*<.05* for FDR corrected values. Only FDR corrected values are interpreted as significant.

Quadratic Age Associations with Network Segregation							
	**Quadratic Age β coefficient**	**Raw *p*-value**	**FDR Corrected *p*-value**	**R^2**	**Adjusted R^2**	**Residual Std. Error (df = 116)**	**F Statistic (df = 2; 116)**
**Au**	-0.0002	0.003	0.017*	0.101	0.085	0.099	6.515
**CBBG**	-0.0003	0.001	0.011*	0.117	0.101	0.157	7.660
**COTC**	-0.002	0.288	0.390	0.045	0.029	3.212	2.741
**DA**	-0.0001	0.236	0.390	0.02	0.003	0.111	1.203
**DM**	-0.0001	0.072	0.198	0.028	0.011	0.142	1.664
**FPTC**	-0.00003	0.637	0.637	0.015	-0.002	0.134	0.897
**Sa**	-0.0001	0.034	0.125	0.063	0.047	0.131	3.932
**SSH**	-0.0001	0.096	0.211	0.03	0.013	0.11	1.805
**SSM**	-0.00004	0.317	0.390	0.036	0.019	0.073	2.148
**Vi**	-0.0001	0.319	0.390	0.034	0.018	0.123	2.061
**VA**	-0.0001	0.472	0.519	0.014	-0.003	0.212	0.842

Supplementary Table 11. Superior model fit would be determined by a difference of |10| between linear and quadratic models. Asterisk indicates superior fit in a quadratic model as compared to a linear model for age and network segregation.

Female Linear and Quadratic Age Relationships with Network Segregation AIC Model Fit Comparisons			
	**Female Linear AIC**	**Female Quadratic AIC**	**Female Difference (Linear AIC-Quad AIC)**
**Au**	-106.723	-111.224	4.502
**CBBG**	-44.239	-53.560	9.321
**COTC**	371.517	372.737	-1.220
**DA**	-105.371	-106.819	1.448
**DM**	-63.105	-65.445	2.339
**FPTC**	-81.679	-83.478	1.800
**Sa**	-67.344	-78.458	11.114*
**SSH**	-96.195	-98.919	2.724
**SSM**	-146.535	-146.629	0.094
**Vi**	-77.789	-77.762	-0.028
**VA**	3.374	5.046	-1.672

Supplementary Table 12. This table exhibits results from linear regressions for hormone interactions and network segregation in males. Raw *p*-values and FDR corrected *p*-values are included. FDR correction was only performed if raw *p* -value was <.05. There were no significant findings after FDR correction.

Male Hormone Level Interactions with Network Segregation											
	**Estradiol by Progesterone** (E*P) ***β* coefficient**	**Raw E*P *p*-value**	**Estradiol by Testosterone** (E*T) ***β* coefficient**	**Raw E*T *p*-value**	**Progesterone by Testosterone** (P*T) ***β* coefficient**	**Raw P*T *p*-value**	**FDR Corrected *p*-value for Estradiol by Progesterone**	**R^2**	**Adjusted R^2**	**Residual Std. Error (df = 39)**	**F Statistic (df = 6; 39)**
**Au**	-0.0004	0.592	0.001	0.299	0	0.634	0.627	0.07	-0.074	0.097	0.486
**CBBG**	-0.001	0.627	0.002	0.463	0	0.955	0.627	0.04	-0.108	0.176	0.272
**COTC**	-0.033	0.041	0.016	0.468	-0.0003	0.123	0.226	0.278	0.167	1.818	2.500
**DA**	-0.001	0.361	0.002	0.242	-0.00002	0.153	0.627	0.072	-0.071	0.127	0.501
**DM**	-0.001	0.549	0.001	0.496	-0.00001	0.603	0.627	0.037	-0.111	0.146	0.25
**FPTC**	-0.003	0.032	0.003	0.113	-0.00001	0.410	0.226	0.168	0.04	0.147	1.309
**Sa**	0.001	0.622	-0.0002	0.924	-0.00001	0.537	0.627	0.024	-0.126	0.142	0.163
**SSH**	-0.001	0.340	0.002	0.252	0	0.752	0.627	0.101	-0.037	0.116	0.733
**SSM**	-0.001	0.187	0.001	0.469	-0.00001	0.251	0.611	0.12	-0.016	0.067	0.884
**Vi**	-0.001	0.222	0.001	0.328	0	0.694	0.611	0.074	-0.068	0.114	0.523
**VA**	-0.001	0.467	0.0004	0.834	-0.00001	0.471	0.627	0.054	-0.092	0.176	0.368

Supplementary Table 13. This table exhibits results from linear regressions for hormone interactions and network segregation in across females and males. Raw *p*-values are included. FDR correction was only performed if raw *p* -value was <.05.

Hormone Level Interactions with Network Segregation										
	**Estradiol by Progesterone** (E*P) ***β* coefficient**	**Raw E*P *p*-value**	**Estradiol by Testosterone** (E*T) ***β* coefficient**	**Raw E*T *p*-value**	**Progesterone by Testosterone** (P*T) ***β* coefficient**	**Raw P*T *p*-value**	**R^2**	**Adjusted R^2**	**Residual Std. Error (df = 94)**	**F Statistic (df = 6; 94)**
**Au**	-0.0001	0.681	-0.0001	0.917	0	0.484	0.059	-0.002	0.1	0.974
**CBBG**	0.001	0.199	-0.0001	0.929	0	0.704	0.045	-0.016	0.163	0.744
**COTC**	-0.016	0.148	-0.015	0.410	0.0001	0.618	0.165	0.111	3.241	3.091
**DA**	-0.0005	0.219	0.0004	0.564	0	0.590	0.041	-0.021	0.116	0.662
**DM**	-0.0002	0.623	0	0.999	0	0.959	0.029	-0.033	0.145	0.461
**FPTC**	-0.001	0.267	-0.0005	0.510	0	0.872	0.091	0.033	0.136	1.561
**Sa**	0.0003	0.490	-0.0004	0.578	0	0.938	0.056	-0.004	0.138	0.936
**SSH**	0.00002	0.964	-0.0002	0.781	0	0.723	0.022	-0.04	0.114	0.357
**SSM**	-0.0003	0.253	0.0001	0.735	0	0.765	0.03	-0.032	0.074	0.486
**Vi**	-0.001[*]	0.084	0.0005	0.483	0	0.917	0.043	-0.018	0.127	0.705
**VA**	0.0001	0.881	-0.0004	0.742	0	0.940	0.004	-0.06	0.226	0.057

Supplementary Figure 1. This table presents locally weighted scatterplot smoothing for each sex steroid hormone and age. The gray superimposed on each colored line depicts the 95% confidence interval in each sex. For comparison of these data, refer to ZRT laboratory services saliva reference range (https://www.zrtlab.com/images/documents/zrt_saliva_reference_range_determination.pdf) which visualizes normative data on relationships between each of these sex hormone levels and age.

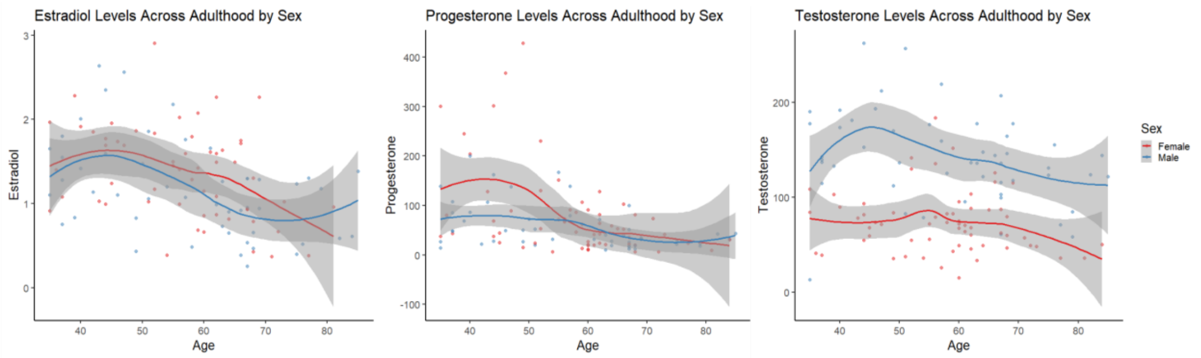

Supplement: Supplementary file 1 [file Data_Sheet_1.pdf]
